# The Effect of Low-Processing Temperature on the Physicochemical and Mechanical Properties of Bovine Hydroxyapatite Bone Substitutes

**DOI:** 10.3390/ma15082798

**Published:** 2022-04-11

**Authors:** Dina Abdelmoneim, Gemma Claire Porter, Dawn Elizabeth Coates, Warwick John Duncan, John Neil Waddell, Niels Hammer, Kai Chun Li

**Affiliations:** 1 Sir John Walsh Research Institute, Faculty of Dentistry, University of Otago, Dunedin 9016, New Zealand; gemmacotton22@gmail.com (G.C.P.); dawn.coates@otago.ac.nz (D.E.C.); warwick.duncan@otago.ac.nz (W.J.D.); j.neil.waddell@icloud.com (J.N.W.); kc.li@otago.ac.nz (K.C.L.); 2 Division of Macroscopic and Clinical Anatomy, Gottfried Schatz Research Center, Medical University of Graz, 8010 Graz, Austria; niels.hammer@medunigraz.at; 3 Department of Orthopedic and Trauma Surgery, University of Leipzig, 04103 Leipzig, Germany; 4 Fraunhofer Fraunhofer Institute for Machine Tools and Forming Technology (IWU), Medical Branch, 01187 Dresden, Germany

**Keywords:** bone grafts, xenografts, resorption, deproteinisation

## Abstract

Bovine bone grafts (BBX) require protein removal as part of the manufacturing process to reduce antigenicity and, in consequence, to be safely used in humans. Deproteinisation may have direct effects on the characteristics of the bone material and on in vivo material performance. This research aimed to comprehensively study the physicochemical and mechanical properties of BBX processed at low deproteinisation processing temperatures. Cubes of bovine bone (8 mm^3^) were treated with temperatures between 100 °C and 220 °C at 30 °C intervals and with pressures ranging from 1.01 to 24.58 Bar. The samples were characterised topographically and mechanically using scanning electron microscopy (SEM), atomic force microscopy (AFM), and uniaxial bending tests. The organic content and the chemical composition were determined using thermogravimetric analysis (TGA) and Fourier-transform infrared spectroscopy (FTIR). X-ray diffraction (XRD) and FTIR were also used to quantitatively determine the specimen crystallinity. Increasing temperature/pressure was associated with decreasing protein levels and compressive strength and increasing surface irregularities and crystallinity. The findings suggest that low-temperature processed bone is likely to exhibit a rapid in vivo degradation rate. The deproteinisation temperature can be adjusted to tailor the graft properties for specific applications.

## 1. Introduction

Vital healthy bone tissues exhibit a certain degree of natural healing; however, large defects due to bony injury following trauma, cancer, or osseous disease can require surgical intervention and bone replacement. First-line treatments for such defects are bone grafting procedures. Bone grafts provide an osteoconductive scaffold to support osseous ingrowth and may also contain osteoinductive substrates for osteoprogenitor chemotaxis and mitogenesis, hence promoting wound healing [1]. An ideal scaffold material should be biocompatible, nonimmunogenic, and cost-effective, possess adequate mechanical strength, and eventually should be replaced by the host’s own bone [2].

Autografts are bone grafts retrieved from the patient undergoing treatment, and can be harvested from the chin, ramus, iliac crest or a variety of other regions [3]. They are considered the gold standard bone replacement graft due to their high clinical success and good regenerative outcomes [4]. However, autograft harvesting procedures are expensive and time-consuming, while also accompanied with a risk of donor site morbidity and pain [5,6,7,8,9]. An alternative source of bone is from xenografts such as bovine bone xenograft (BBX) derived from cattle bone. Use of BBX is widely recognised and has been proven to be safe for a number of human applications [10]. BBX has advantages over autografts as they can be mass-produced at a reasonable cost, are abundant in supply, and do not require additional surgery for sample retrieval. Furthermore, BBX has a similar morphological structure and physicochemical properties when compared to human cancellous bone [11,12], making it a favourable human bone substitute material.

Bone is a composite tissue composed of an organic matrix and inorganic minerals [13]. The organic matrix is composed mainly of collagen, whereas the inorganic phase consists of calcium, phosphorous, and oxygen, combined to form crystalline hydroxyapatite (HA). As BBX is sourced from nonhuman species, the collagen and other protein constituents of bone present a risk of cross-species immunogenicity and disease transmission such as bovine bone encephalopathy, necessitating a protein removal step (deproteinisation and sterilisation) as part of the manufacturing in order to meet regulatory approval for human applications. A common approach for deproteinisation involves heat treatment, which has a direct effect on the physicochemical and mechanical characteristics of BBX, such as porosity, surface roughness, crystallinity, and mechanical strength [14].

Changes in the physicochemical characteristics of bone graft materials may influence their handling properties, resorption, and role in osteogenesis. The manufacturing process can be manipulated to alter the grafts’ characteristics, thereby optimising them to suit a specific application. The bone scaffold should be designed in a way that degradation matches the osteogenic synthesis rate. Neither slower nor faster resorption rates of the grafting material seem desirable, as both can negatively affect the healing outcome. Ideally, a scaffold would retain its physical properties for a minimum duration of 1–3 months [15], thereby providing mechanical stability. Tadic and Maple (2004) compared 14 commercially available bone grafting materials and found that increased manufacturing temperatures between 300 °C and 1200 °C enhanced material crystallinity [16]. On the other hand, crystallinity and temperature exposure while manufacturing are inversely proportional to the rate of resorption [17]. Thus, one method to decrease the resorption rate is to heat-treat the bone substitutes at a high temperature.

Bio-Oss^®^ (BO) (Geistlich Pharma AG, Wolhusen, Switzerland) is a widely used commercially available BBX in a granular form obtained by chemically treating degreased bovine bone followed by heat treatment at 350 °C [18]. The success of bone grafting when combined with dental implant placement greatly depends on osteointegration, the formation of a direct structural and functional connection between living bone and the surface of the implant [19]. However, in previous studies, BO has been associated with connective tissue encapsulation and a slow rate of resorption, which can interfere with both new bone formation and osteointegration [20,21,22,23]. MoaBone^®^ (MB) (Molteno Ophthalmic Ltd., Dunedin, New Zealand), another BBX material, is derived from New Zealand (NZ) prion-free cattle. There has never been a case of bovine spongiform encephalopathy in NZ. MB has been widely used for ophthalmic grafting [24], but has not been investigated in oral applications. According to the manufacturer, MB is produced through heating and degreasing cattle bone at about 80 °C followed by chemical treatment. Smith et al. reported the strong activation of osteoclasts with MB compared to BO, which resulted in similar rates of osseointegration and no signs of an inflammatory response [25]. This indicates the strong potential for MB to be a grafting material, where complete remodelling is desired. However, in its currently available form, MB has a high resorption rate which limits its applications. Modifications to the material processing of MB via thermal deproteinisation may alter its physicochemical characteristics to produce properties consistent with lower-resorption-rate materials (i.e., high crystallinity, low organic content).

The performance of the bone grafting substitutes principally depends on the materials’ morphology and composition. Therefore, the purpose of this study was to establish the effect of five low deproteinisation temperatures (100 °C, 130 °C, 160 °C, 190 °C, 220 °C) on the properties of MB, referred to as MB 100 °C, MB 130 °C, MB 160 °C, MB 190 °C and MB 220 °C, respectively. The chosen temperatures lie between the deproteinisation temperatures of the commercial MB and BO. Surface topography, chemical composition, crystallinity and mechanical properties were investigated and compared to the MB pre-treatment referred to as half-processed MB (HP-MB), fully processed MB as supplied by the manufacturer, and BO.

## 2. Materials and Methods

The following materials were used in the study; Bio-Oss^®^ (BO) (Cat no: 20112, Geistlich Pharma, Switzerland), full-processed MoaBone^®^ as supplied by the manufacturer (MB) (MOLTENO Ophthalmic Ltd., Dunedin, New Zealand), half-processed MoaBone (HP-MB) (degreased), batch no. 1810/SBE2 and heat-treated MB prepared from the HP-MB (HT-MB) as described in the next section.

### 2.1. Sample Preparation and Heat Treatment

Cubes of HP-MB measuring 25 × 25 ×25 mm^3^ which had undergone initial degreasing were supplied by MOLTENO Ophthalmic Ltd. Randomly selected cubes were further processed into smaller 8 × 8 × 8 mm^3^ cubes or sectioned into 2 mm slices using a precision cutting machine (Accutom-50, Struers, Ballerup, Denmark). The thermal treatment setup consisted of a custom-built cylindrical stainless-steel heating vessel with an external diameter of 120 mm, internal diameter of 70 mm and height of 105 mm, fitted with a pressure gauge to record the increase in pressure commensurate with the increase in temperature. The 8 mm^3^ cubes and slices of bone were randomly allocated to their sample groups based on treatment temperature prior to being placed in the stainless-steel vessel containing distilled water (water:bone ratio was at least 1:1), heated at a rate of 4–6 °C/min and held for 2 h at the following temperatures: Group MB 100 °C-100 °C, Group MB 130 °C-130 °C, Group MB 160 °C-160 °C, Group MB 190 °C-190 °C and Group MB 220 °C-220 °C, resulting in five groups of heat/pressure-treated MoaBone collectively referred to as HT-MB. The pressure was read directly from an attached pressure gauge and verified against the Clausius–Clapeyron relation, as presented in Table 1. On completion of the thermal cycle, the vessel was then cooled in 15 °C tap water for 5 min prior to removal of the bone specimens. Bone specimens were rinsed with distilled water and air-dried for 24 h under sterile conditions before testing.

### 2.2. Scanning Electron Microscopy (SEM) Imaging

Prior to SEM imaging, specimens (n = 3 per group) were sputter-coated with a 10 nm gold-palladium layer (Emitech K575X, EM Technologies Ltd., Kent, UK). The surfaces were analysed under 90× and 30,000× magnification with a field-emission SEM (Joel 6700F, Joel Ltd., Tokyo, Japan) using the secondary electron imaging mode @ 10 kV accelerating voltage. Representative regions were described and compared. ImageJ was used to measure the average diameter of the pores size. Three images at 90× were assessed per specimen. Randomly selected pores were analysed for the short pore axis.

### 2.3. Surface Topography Imaging Using Atomic Force Microscopy (AFM)

Heat-treated specimen slices measuring 2 mm in thickness (n = 3) were prepared at three different temperatures (low = 100 °C, medium = 160 °C, high = 220 °C) to determine the differences in surface topography for the HT-MB specimens and compared against BO. The HT-MB slices and BO granules were affixed to a standard glass microscope slide (Thermofisher, Waltham, MA, USA) and AFM measurements were conducted using a Bioscope catalyst atomic force microscope (Bruker, Billerica, MA, USA). The imaging was performed using a silicon probe (RTESPA-150, Bruker, Billerica, MA, USA) with a spring constant of 5 N/m and resonant frequency of 150 kHz. The images were generated using the PeakForce Quantitative Nanoscale Mechanical Characterisation mode in air, at room temperature with a scan rate of 0.5–1 Hz and a grid size of 20 × 20 µm^2^. Low-density polyethylene polymer (PS-LDPE-12M, Bruker, Billerica, MA, USA) was used as a reference standard. Nanoscope software 9.4 (Bruker, Billerica, MA, USA) was used to calculate the surface roughness and elastic modulus.

### 2.4. Compressive Strength

Heat-treated specimen cubes measuring 8 × 8 × 8 mm^3^ (n = 30) were prepared at three different temperatures (low = 100 °C, medium = 160 °C, high = 220 °C) to determine their compressive strength. The compressive strength test was conducted with a universal testing machine (Instron 3369 Universal Testing machine, Norwood, MA, USA) equipped with a 500 N load cell at a crosshead speed of 2 mm/min. Weibull analysis of the compression data was conducted to calculate the Weibull modulus and the normalising strength of each group of materials.

### 2.5. Determining the Residual Organic Content by Thermogravimetric Analysis (TGA)

The residual organic contents of all HT-MB groups, HP-MB, MB and BO (n = 2), were determined using a *Q50* Thermogravimetric Analyser (TA instruments, New Castle, DE, USA). The specimens were ground into a fine powder and 20 to 25 mg was placed onto a platinum tray. The thermal decomposition was carried out in air at room temperature up to 1000 °C with a heating rate of 20 °C/min. The process was repeated twice and presented as an average of both runs.

### 2.6. Chemical Composition and Crystallinity Analysis by Fourier-Transform Infrared Spectroscopy (FTIR)

The chemical analysis was performed with an attenuated total reflectance FTIR spectrometer (Alpha II, Bruker, Germany). Samples (n = 2) of all the HT-MB groups, HP-MB, MB and BO, were analysed. The spectra were recorded in the range of 4000–400 cm^−1^, at a resolution of 4 cm^−1^, with a total of 24 scans per run. The baseline was corrected for all the specimens. Each sample and control were repeated twice and presented as an average of both runs. The crystallinity index (C.I) was calculated according to Weiner and Bar-Yosef (1990) using the following formula C.I = (A + C)/B [26]. The heights of the absorption bands at 603 cm^−1^ (referred to as A) and 565 cm^−1^ (referred to as C) were summed and divided by the height of the valley between the two bands (referred to as B). The mineral content:organic matrix (M:M) was calculated as the phosphate-to-amide I ratio, and the relative carbonate content was calculated as the carbonate-to-phosphate ratio (C:P) [27]. The M:M was determined by calculating the ratio of the peak area assigned to phosphate bands at 900–1200 cm^−1^ to the peak areas of 1585–1720 cm^−1^ assigned to Amide I, while the C:P was determined by calculating the ratio of the carbonate peak area at 1405 cm^−1^ to the phosphate band area at 900–1200 cm^−1^.

### 2.7. Crystallinity Analysis by X-ray Diffraction

The crystallinity of bone specimens was determined using powdered XRD (X’Pert Pro MPD, Malvern Panalytical, Malvern, UK). Samples (n = 3) of all the HT-MB groups, HP-MB, MB and BO, were ground into fine powder using an agate mortar and pestle, packed into a specimen holder, and measured with the diffractometer at scan steps of 0.05°. The diffractometer operated at 40 kV and 30 mA, using Cu K_α_ radiation with a scan range of 20–80° 2 *θ*. Two methods were used to calculate the crystallinity. In method 1, the degree of crystallinity was calculated using Origin Lab Pro 2008 software considering the whole diffractogram by subtracting the crystalline peaks from the total area of the graph (i.e., crystalline and amorphous area). Method 2 was adapted from Landi, Tempieri, Gelotti, and Sprio (2000) where the crystalline phase was evaluated by the relation: X_c_ ≈ 1 − (V_112/300_/I_300_), where I_300_ is the intensity of 300 reflection and V_112/300_ is the intensity of the hollow between 112 and 300 reflections [28].

### 2.8. Statistical Analyses

The mean ± standard deviation was calculated for the crystallinity from XRD data (n = 3), compressive strength (n = 30), surface roughness (n = 3), and elastic modulus (n = 3). The TGA data and the C/P ratio, M:M ratio and C.I index (calculated from the FTIR data) were presented as a mean of two runs. All data were analysed using GraphPad Prism 7. One-way ANOVA was used for group comparisons of the crystallinity obtained from the XRD data, elastic modulus and surface roughness data. For compression strength analysis, the Kruskal–Wallis test was used for group comparisons. *p* ≤ 0.05 was considered as statistically significant.

## 3. Results

### 3.1. Surface Morphology of Bovine Bone Graft Materials as Determined by SEM

**Figure 1:**
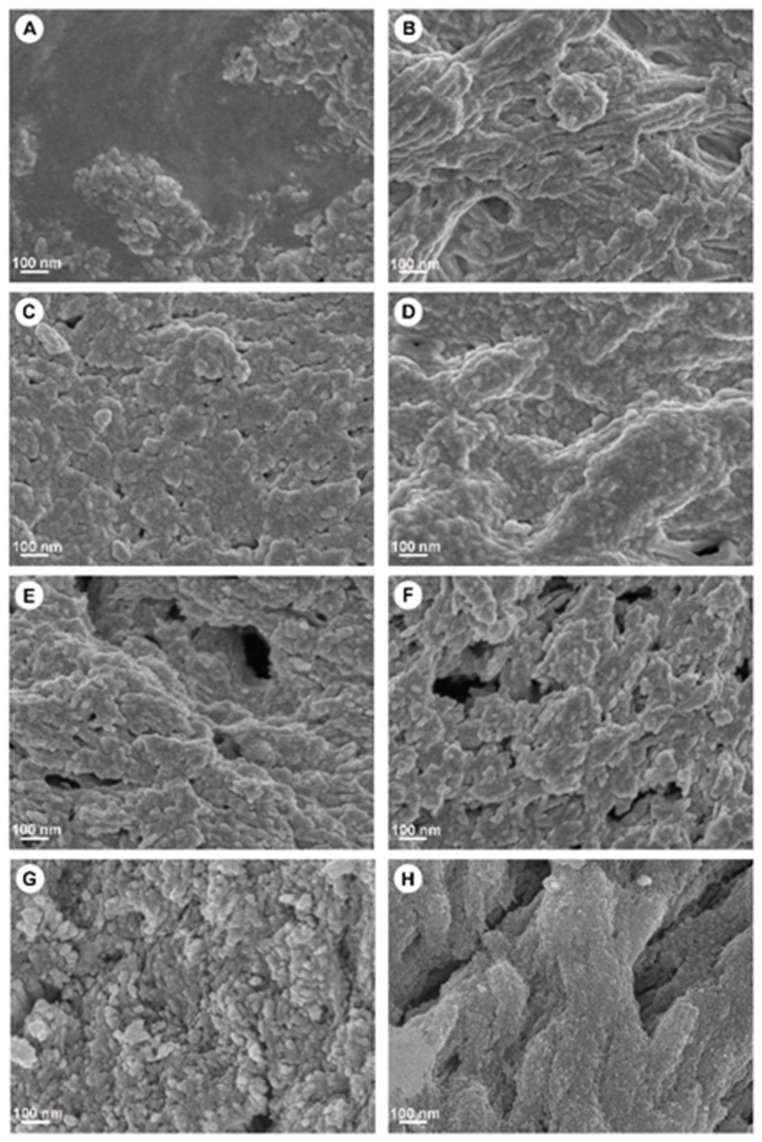
Representative scanning electron microscope images of the surface of heat-treated MoaBone and controls. (**A**) Half-processed MB, (**B**) MB 100 °C, (**C**) MB 130 °C, (**D**) MB 160 °C, (**E**) MB 190 °C, (**F**) MB 220 °C, (**G**) MoaBone^®^, (**H**) Bio-Oss^®^. Scale bar = 100 nm.

SEM was used to study the microscopic changes of the bone surface associated with the different heat temperatures. Figure 1 shows representative SEM images of the HT-MB groups, HP-MB, MB, and BO at 30,000× magnification. The HP-MB samples appeared to be covered with a smooth wax-like structure, which was not observed across any of the other samples. MB 100 °C had a fibrous-like structure, whereas samples prepared at 130 °C, 160 °C, 190 °C and 220 °C were characterised by irregular surfaces; as the temperature increased, the surface irregularity increased. The BO surface was characterised by high elevations and deep depressions, which was not observed in any of the other HT-MB samples. The pore sizes ranged from 100 to 650 µm. Representative low-magnification images are presented in the Supplementary Materials, Figure S1.

### 3.2. Surface Topography and Mechanical Properties Established by AFM

**Figure 2:**
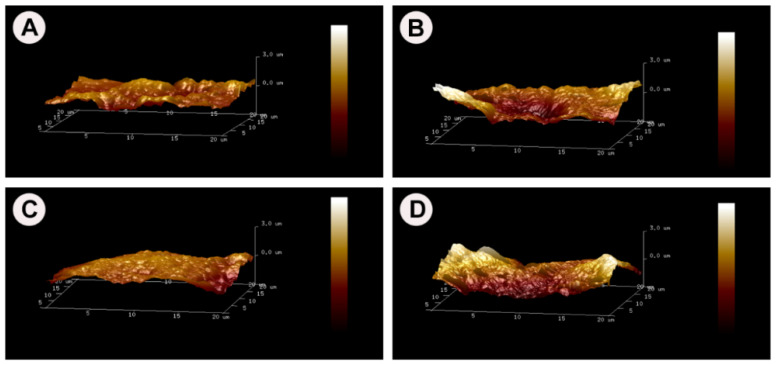
Atomic force microscopy 3D height images of the heat-treated MoaBone and Bio-Oss^®^. (**A**) MB 100 °C, (**B**) MB 160 °C, (**C**) MB 220 °C and (**D**) Bio-Oss^®^.

**Figure 3:**
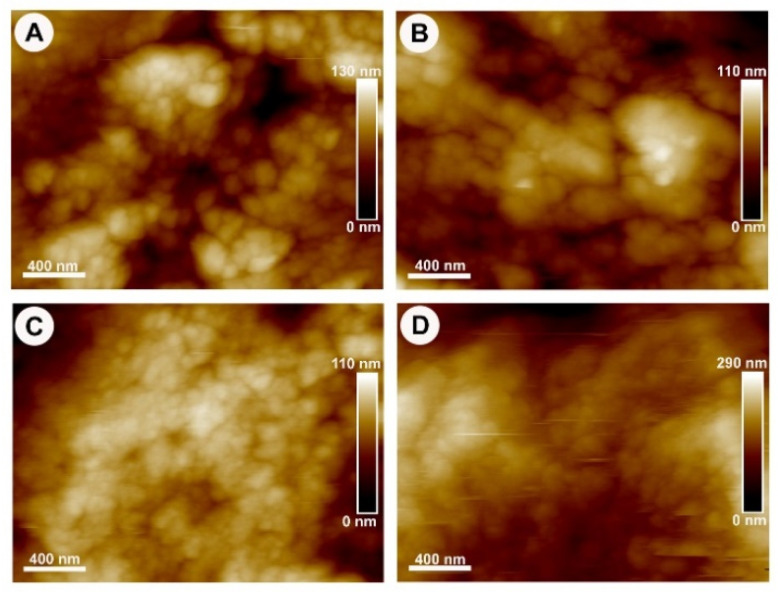
Atomic force microscopy 2D-height topography images of the heat-treated MoaBone and Bio-Oss^®^. (**A**) MB 100 °C, (**B**) MB 160 °C, (**C**) MB 220 °C and (**D**) Bio-Oss^®^. Scale bar = 400 nm.

**Figure 4:**
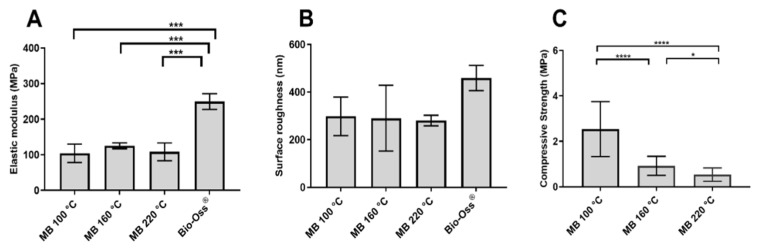
Graphs showing the mechanical properties. (**A**) Elastic modulus of the heat-heated MoaBone processed at 100 °C, 160 °C, 220 °C and Bio-Oss^®^ calculated from atomic force microscopy. (**B**) Surface roughness of the heat-heated MoaBone processed at 100 °C, 160 °C, 220 °C and Bio-Oss^®^ calculated from atomic force microscopy. (**C**) Compressive strength of heat-treated MoaBone processed at 100 °C, 160 °C and 220 °C. Mean ± SD. * *p* < 0.05, *** *p* ≤ 0.0005, **** *p* < 0.0001.

The surface topography of MB 100 °C, MB 160 °C, MB 220 °C, and BO as demonstrated by AFM is summarised in Figure 2 (3D image) and Figure 3 (2D image) as color-coded height images, where dark pixels are considered low points and light pixels are considered high points. The three HT-MB groups and BO specimens showed a crystalline structure with crystals measuring 50–200 nm rather than the typical collagen banding pattern of bone. In the AFM load displacement measurements (Figure 4A), BO had a significantly higher elastic modulus with a mean of 199 MPa compared to all the HT-MB samples (*p* ≤ 0.0005). The mean elastic modulus was 104, 125 and 108 MPa for MB 100 °C, MB 160 °C and MB 220 °C, respectively, with no statistically significant difference found among the three HT-MB samples. The value R_a_ reflects the arithmetic mean of the absolute values of the surface point departures from the mean plane within the sampling area [29]. Figure 4B shows the surface roughness calculated for the three HT-MB groups and BO. No statistically significant difference was found across the test and control samples. BO had the highest surface roughness with a mean R_a_ value of 459 nm, and all the three HT-MB groups had similar mean R_a_ values of 298, 290 and 280 nm for MB 100 °C, MB 160 °C and MB 220 °C, respectively.

### 3.3. Compressive Strength

Compressive strength was measured for MB 100 °C, 160 °C and 220 °C (Figure 4). MB processed at 100 °C had the strongest compressive strength ranging between 0.47 and 5.13 MPa with a mean of 2.4 MPa (±1.19), which was significantly higher than those of the other test samples (*p* < 0.0001). Treatment at 160 °C gave a compressive strength of 0.40–1.9 MPa with a mean of 0.93 MPa (±0.4), and this was significantly higher than MB at 220 °C (*p* = 0.0117). The lowest compressive strengths were measured on the bone samples processed at 220 °C, ranging between 0.19 and 1.28 MPa with a mean of 0.53 MPa (±0.29). Weibull analysis of the compression data was conducted to calculate the Weibull modulus and the normalising strength of each group. The normalising strength averaged 2.80, 1.04 and 0.60 MPa, with Weibull moduli of 2.22, 2.71 and 2.18 MPa for MB 100 °C, 160 °C and 220 °C, respectively.

### 3.4. Residual Organic Content

**Figure 5:**
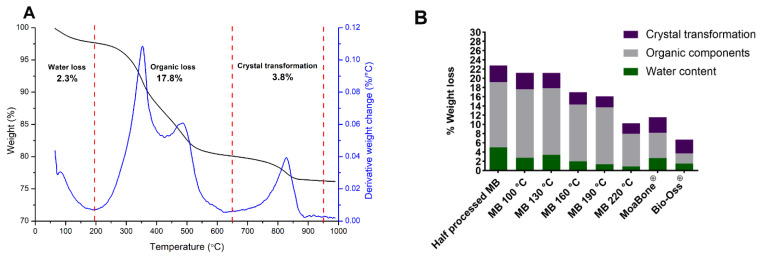
(**A**) Representative TGA graph of the half-processed MoaBone showing percentage weight loss in correspondence to temperature (black), and derived weight change (blue): Water loss between room temperature to 195 °C; Organic loss between 195 °C and 650 °C; Crystal transformation between 650 °C and 950 °C. (**B**) Graph showing the percentage weight loss of the water content, organic components and crystal transformation calculated for the half-processed MoaBone, heat-treated MoaBone, MoaBone^®^ and Bio-Oss^®^ from an average of two replicates.

All the HT-MB groups, HP-MB, MB and BO, were analysed by TGA to determine the remaining organic materials. A representative TGA curve of the HP-MB is presented in Figure 5A. The TGA curve corresponds to percentage weight loss over time with respect to temperature change, as shown in black in Figure 5A. The derived weight change is plotted against temperature and shown in blue, referring to the rate of weight changes to help determine the temperature ranges of the major weight drops. Consistent with the literature, three major weight losses were observed. An initial weight loss was observed up to 195–200 °C due to the evaporation of water, followed by a major weight loss between 200 °C and 650 °C, which corresponds to the burning of the remaining organic components such as fats and collagen [30]. A small weight loss was detected at 650 °C to 950 °C owing to the decomposition of the carbonated minerals (carbonated apatite) [30] and the transformation of hydroxyapatite crystals [31]. In general, bone graft materials treated at higher temperatures (above 130 °C) had decreased levels of organic content when compared to MB prepared at lower temperatures (Figure 5B). BO had the least organic loss of 2.2%, followed by MB with an organic loss of 5.5%, and HP-MB had the highest organic loss of 14.15%.

### 3.5. Chemical Composition and Crystallinity by FTIR

**Figure 6:**
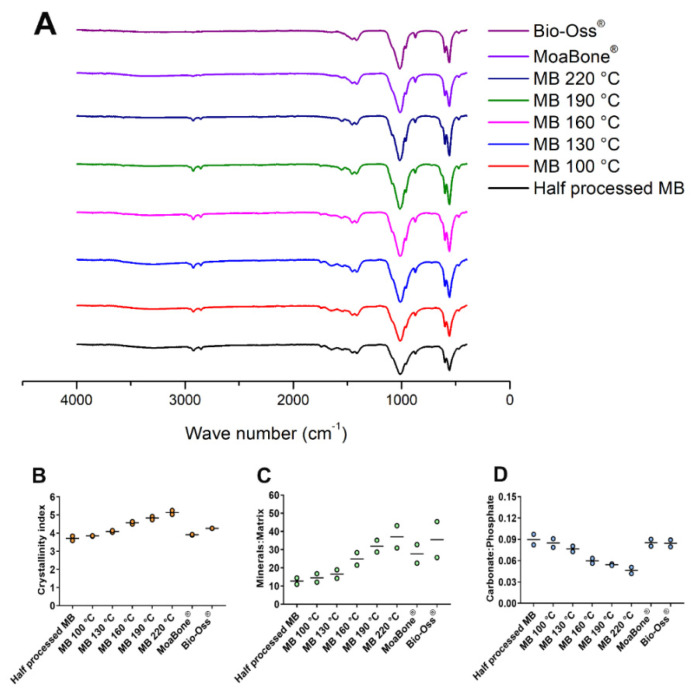
(**A**) Fourier-transform infrared spectroscopy spectra of the half-processed MoaBone, heat-treated MoaBone, MoaBone^®^ and Bio-Oss^®^. Graphs showing bone composition properties calculated from the Fourier-transform infrared spectra for the half-processed MoaBone, heat-treated MoaBone, MoaBone^®^ and Bio-Oss^®^. (**B**) Crystallinity index. (**C**) Minerals:matrix ratio. (**D**) Carbonate:phosphate ratio. Line = mean.

FTIR was used to identify the chemical composition and functional groups in all the HT-MB groups, HP-MB, MB and BO. The test samples and the controls exhibited spectra (Figure 6A) similar to the BBX spectra reported in the literature [13,15,31,32,33,34,35,36,37]. Phosphate bands were detected in the range between 470–600 cm^−1^ and 960–1013 cm^−1^. These bands had higher intensities with increasing temperatures. Similarly, the calculated C.I also increased with the temperature (Figure 6B). Collagen-specific amide bands Amide I (C = O stretching), Amide II and Amide III (N = H deformation) were identified at 1650–1740, 1541 and 1252 cm^−1^, respectively. The Amide I peak was more evident in the HP-MB and in HT-MB treated at lower temperatures, which was also indicated by the lower M:M ratios (Figure 6C). A double band at 1416 and 1456 cm^−1^, corresponding to the stretching vibrations of CO_3_ (B-type) substituting for phosphate in the apatite lattice, was observed in all samples and found to be more distinct in MB and BO [35]. The relative carbonate content decreased upon increasing the bone processing temperature (Figure 6D). A broad band in the range of 3000–3500 cm^−1^, derived from the OH-bond of water, was visible in all samples, and decreased in intensity with the temperature. The spectra of the HT-MB had an additional two bands at ~2851 and 2921 cm^−1^, which were derived from the C–H stretching of the remaining organic components.

### 3.6. Crystallinity Utilising XRD

**Figure 7:**
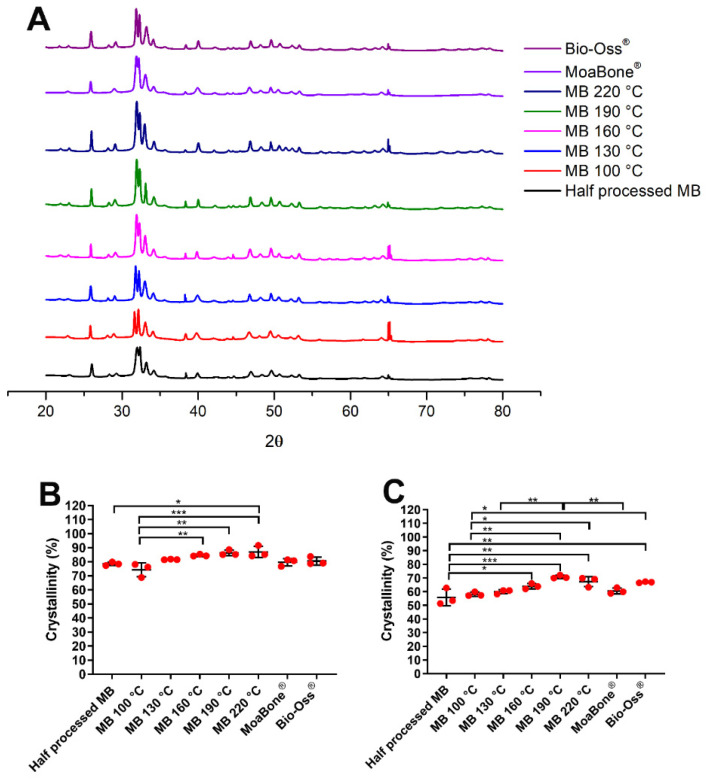
(**A**) X-ray diffraction spectra of the half-processed MoaBone, heat-treated MoaBone, MoaBone^®^ and Bio-Oss^®^. Bottom: Graphs summarising crystallinity of the half-processed MoaBone, heat-treated MoaBone, MoaBone^®^ and Bio-Oss^®^. (**B**) Method 1, (**C**) Method 2. Mean ± SD. * *p* < 0.05, ** *p* ≤ 0.001, *** *p* ≤ 0.0001.

The XRD data are presented as a peak diffractogram; narrow and sharp peaks are an indication of a highly crystalline material, whereas broader peaks indicate low crystallinity. The diffraction patterns of hydroxyapatite were the only crystalline phase detected in the prepared powders. The XRD spectra of the powders are shown Figure 7A. As the temperature increased, the resultant XRD peaks became sharper, indicating an increase in crystallinity. XRD spectra were utilised to quantify the materials’ crystallinity using two methods. Figure 7B,C show the average crystallinity calculated via the two methods previously described for each temperature-processed bone group. The two methods showed similar trends, and as the temperature increased, crystallinity increased. MB 220 °C had the highest crystallinity of 87% for method 1, while MB 190 °C had the highest crystallinity of 70% for method 2. MB 100 °C and HP-MB had the least crystallinity using method 1 and 2, respectively.

## 4. Discussion

Biological bone substitutes require deproteinisation to prevent any immunological or pathological reaction that would compromise safety when used in clinical applications [32]. A commonly utilised method for deproteinisation is heat-treatment, which has been found to influence the physical, chemical, biological and mechanical properties of the bone, and hence the clinical outcomes during osseous healing [33,34,35]. We investigated the effects of five deproteinisation temperatures upon the physicochemical properties of MB, and compared these to BO, a commonly used BBX grafting material. As the applied temperature increased, we observed an increase in surface irregularities on the grafting material and saw evidence of increasing crystallinity, along with a concomitant decrease in organic content and compressive strength.

### 4.1. Changes in Surface Texture and Topography with Increased Temperature

Surface properties such as topography and surface chemistry are considered a determining factor for cellular adherence and therefore contribute greatly to the host tissue response. Rough surfaces are believed to enhance contact osteogenesis at the early stages of healing by promoting osteoconduction [36]. Topographically complex surfaces promote osteoconduction by increasing the surface area for fibrin attachment and providing surface features, allowing for fibrin entanglement [37]. The ‘roughness average’ known as R_a_ is commonly used to quantify the surface roughness of biomaterials. Biomaterials with high R_a_ values have been associated with enhanced bone-to-material contact and increased new bone formation [38]. In a study that investigated bone formation on titanium surfaces rather than bone, an increased surface roughness of titanium surfaces demonstrated a significant increase in osteoblastic attachment and proliferation with R_a_ values of 53.25 ± 1.03 nm, compared to smooth surfaces with R_a_ of 4.87 ± 1.39 nm [29]. Additionally, an increase in alkaline phosphatase (ALP) enzyme, which is an indicator for osteogenic differentiation, has been previously reported when surface roughness increases [39]. In our study, as the applied temperature increased, SEM imaging of the treated bone surfaces showed increases in surface irregularities. However, at the nanoscopic level, the surface roughness data collected from the AFM images did not support an increased roughness across the tested temperatures. At very high magnifications, differences may have been observed, but this was not possible in our current study, due to limitations in the current AFM capability and the surface irregularity of the bone being analysed. The obtained R_a_ values of both the HT-MB tested groups and BO suggest that these materials are good substrates for osteoblast attachment.

### 4.2. Relation between Bone Composition and Mechanical Properties

This study examined the relation between mechanical properties and deproteinisation temperature. AFM revealed that the heat treatment had no effect on the elastic modulus of the MB. We found that BO had a significantly higher elastic modulus compared to the three tested HT-MB groups. On the other hand, the heat treatments affected compressive strength; a significant drop in strength was observed comparing MB 100 °C and MB 220 °C specimens. As previously mentioned, bone is a multiphase material and the interaction between the flexible organic matrix-collagen and the stiffer and tougher inorganic phase determines its mechanical properties [34]. Therefore, the denaturation of the collagen would result in significant loss of bone strength with little effect on the stiffness (i.e., elastic modulus). MB 220 °C had the least organic content; therefore, it was expected to have the lowest mechanical strength, with values less than 1 MPa. Similar results were observed by Wang et al. (2001), who reported a significant decrease in bone mechanical properties after bone was heated between 150 °C and 200 °C without a noticeable change in the elastic modulus [35]. The Weibull modulus is usually calculated for brittle materials and indicates variability in the material’s strength, whereas the normalising strength is a measure of the expected stress a material can withstand before fracture. The Weibull parameters showed that MB 160 °C was the most predictable of the three tested temperatures, as a high Weibull modulus indicates that failure would be more likely to be close to the expected normalising strength.

### 4.3. Effect of Temperature on the Organic Component

SEM imaging showed a reduction in fibrous materials and smoother surface coatings with increased temperatures. These surface coatings were similar to the collagen structure previously observed on a commercial deproteinised porcine bone—Gen-Os^®^ processed at 130 °C [40]. The loss of organic matter as suggested by SEM imaging was also observed through TGA analysis. The amount of the remaining organic material is crucial as it determines the ability to safely use the grafting materials without provoking any immunological reactions [32]. Peters et al. performed mass spectrometric analysis of the released compounds of heated bone at the three points of major weight loss. They reported that the major weight loss occurring between 200 °C and 650 °C corresponded to the combustion of the remaining organic content [30]. In our study, TGA analysis of the MB 220 °C showed an additional 7% loss of the remaining organic content compared to the HP-MB. Even though the commercial MB samples were deproteinised at a lower temperature, the remaining organic content was 1.5% higher in the MB 220 °C specimens. This may be due to chemical treatments involved in the final stages of preparing the commercial product. It is also worth noting that the terminal sterilisation with autoclaving and gamma radiation can both result in further denaturation of the collagen structure [41], neither of which was utilised in our study. Collagen type I is the main structural protein in bone and AFM can be used to study structural changes to the collagen fibrillar network in bone. Our AFM images did not show the classic bone appearance which is typically composed of collagen fibrils ranging between 67 and 100 nm in diameter densely coated with mineral platelets [42,43]. The crystalline appearance shown here can be attributed to the deformation of lamellar configuration during the heating process, leaving behind a highly dense mineral content.

### 4.4. Effect of Temperature on the Inorganic Component

The term crystallinity is used interchangeably to describe a material’s properties related to its crystal size, and crystal order structure; high crystallinity values indicate large and ordered crystals, whereas low values correspond to poorly ordered lattices [44]. Structural regularity and large crystal size have been reported in association with an enhanced bone-inducing ability [45]. In that sense, HA crystallinity can be an indicator for bone quality. Using FTIR, the crystallinity can be determined by calculating the C.I, also known as infrared splitting factor (IRSF). The C.I quantifies the splitting extent of the two main peaks of phosphate (PO_4_) groups at 603 cm^−1^ and 565 cm^−1^. The PO_4_ peak sharpening is proportional to the material’s crystallinity (i.e., a more crystalline HA will be characterised by sharper peaks and a higher C.I). FTIR has been shown to be an accurate technique for determining the crystallinity at low heat treatment temperatures. However, high temperatures induce crystal transformation from HA to the β-three-calcium-phosphate phase (β-TCP), and the presence of such mineral phases in the structure may alter the shape of PO_4_ bands, which, in turn, alters the C.I values [46]. Therefore, we determined the crystallinity using two different techniques: XRD and FTIR.

Both techniques have been used extensively to calculate the C.I; however, the values obtained from XRD data cannot be directly compared to those obtained using FTIR. While FTIR depends on the functional groups in the material, XRD calculates the average crystal dimensions to determine the C.I [47]. We also applied two different methods to calculate crystallinity from the obtained XRD data; the first approach takes into account the whole diffractogram (referred to as method 1), whereas the second approach depends on measuring specific peaks (referred to as method 2). This explains the higher crystallinity values obtained from Method 1 compared to Method 2 (i.e., MB 100 °C had 74.4% crystallinity using method 1 compared to 58.1% using method 2). Generally, both the FTIR and the XRD data showed a direct relation between the temperature and the crystallinity values. Our FTIR data showed that the increase in temperature resulted in a higher C.I of 5.14 at 220 °C; meanwhile, HP-MB was characterised by a lower C.I value of 3.71. Similarly, the percentage crystallinity from method 1 showed that the increase in temperature resulted in a higher crystallinity of 87% at 220 °C, compared to 78.5% for HP-MB.

### 4.5. Effect of Temperature on the Atomic Substitution and Bone Composition Properties

A C/P ratio represents the amount of carbonate substitution into the phosphate and hydroxyl positions in the HA lattice [48]. C/P for the commercial xenografts BO and Gen-Oss reported in the literature was 0.12 and 0.57, respectively [49]. An increase in C/P ratio has been linked to increased bone dissolution and osteoclastic resorption [50,51]. The higher solubility of the carbonate-substituted HA was attributed to the weaker Ca-CO_3_ bonds compared to the Ca-PO_4_ [52]. Additionally, increasing carbonate content has also been associated with a decrease in crystal size and increase in micro-strain [53]. Ruppel et al. (2008) reported an inverse relation between the C/P ratio in the crystal lattice and crystallinity [54]. In that context, highly crystalline materials are expected to have large, ordered crystals which are more stable due to the lower carbonate substitution. The relative mineralisation of the collagen matrix (i.e. M:M ratio) increased with temperature. There is also a correlation between collagen denaturation and crystallisation, as removal of the collagen network influences crystallite size, morphology and stoichiometry [33]. The XRD findings supported our FTIR data, where bone treated with lower temperatures such as MB 100 °C was characterised by broader peaks, and thus could be considered less crystalline with a higher organic composition. Rammelt et al. (2004) showed that the addition of collagen to HA promoted both phagocytotic and osteogenic processes compared to plain HA [55]. These findings suggest that lower-temperature-processed bone is likely to be rapidly resorbed in vivo.

## 5. Conclusions

In order to be safe for use in human applications, bone grafts sourced from animal origin undergo a protein removal step as part of the manufacturing process. A common approach for deproteinisation involves thermal processing, which significantly modifies the characteristics of the graft material. Commercially available bovine bone xenografts (BBX) are processed at temperatures ranging between 300 and 1200 °C. Higher processing temperatures have been associated with increased crystallinity and slower resorption rates during osseous healing. We deproteinised a novel BBX MoaBone (MB) under pressure and over a range of low temperatures and compared the physicochemical characteristics with a market-leading natural bovine bone graft, BioOss (BO). Even slight increases in the deproteinisation temperature of BBX significantly modified the physicochemical and mechanical properties. Based on our findings, we hypothesised that heat-treating MB at temperature between 130 °C and 160 °C produces a grafting material with acceptable mechanical properties, resorption rates and surface characteristics. Further in vitro studies are required to assess osteogenic cell attachment and proliferation, followed by in vivo quantification of immunogenicity, graft resorption, and new bone formation in appropriate animal models.

## Figures and Tables

**Table 1:** Pressure recorded at each thermal bone-processing temperature within the stainless-steel vessel.

Temperature (°C)	Pressure (Bar)
100	1.01
130	2.69
160	6.22
190	12.93
220	24.58

## Data Availability

The data presented in this study are available upon request from the corresponding author.

## Supplementary Materials

The following supporting information can be downloaded at: https://www.mdpi.com/article/10.3390/ma15082798/s1, Figure S1: Representative low magnification scanning electron microscope images of the surface of heat-treated MoaBone and controls. (A) Half-processed MB, (B) MB 100 °C, (C) MB 130 °C, (D) MB 160 °C, (E) MB 190 °C, (F) MB 220 °C, (G) MoaBone®, (H) Bio-Oss®. Scale bar = 100 μm; Figure S2: Representative FTIR spectra of the half-processed MoaBone with the relevant characteristic bands labelled.

## References

[1] Schmidt A.H. (2021). Autologous bone graft: Is it still the gold standard?. Inj. Int. J. Care Inj..

[2] Ghassemi T., Shahroodi A., Ebrahimzadeh M.H., Mousavian A., Movaffagh J., Moradi A. (2018). Current concepts in scaffolding for bone tissue engineering. Arch. Bone Jt. Surg..

[3] Kumar P., Vinitha B., Fathima G. (2013). Bone grafts in dentistry. J. Pharm. Bioallied Sci..

[4] Zhao R., Yang R., Cooper P., Khurshid Z., Shavandi A., Ratnayake J. (2021). Bone grafts and substitutes in dentistry: A review of current trends and developments. Molecules.

[5] Swan M.C., Goodacre T.E.E. (2006). Morbidity at the iliac crest donor site following bone grafting of the cleft alveolus. Br. J. Oral Maxillofac. Surg..

[6] Sasso R.C., Williams J.I., Di Masi N., Meyer P.R. (1998). Postoperative drains at the donor sites of iliac-crest bone grafts—A prospective, randomized study of morbidity at the donor site in patients who had a traumatic injury of the spine. J. Bone Jt. Surg..

[7] Castiglioni S., Cazzaniga A., Locatelli L., Maier J.A.M. (2017). Silver nanoparticles in orthopedic applications: New insights on their effects on osteogenic cells. Nanomaterials.

[8] Kalk W.W., Raghoebar G.M., Jansma J., Boering G. (1996). Morbidity from iliac crest bone harvesting. J. Oral Maxillofac. Surg..

[9] Dimitriou R., Mataliotakis G.I., Angoules A.G., Kanakaris N.K., Giannoudis P.V. (2011). Complications following autologous bone graft harvesting from the iliac crest and using the RIA: A systematic review. Injury.

[10] Wu J., Li B., Lin X. (2016). Histological outcomes of sinus augmentation for dental implants with calcium phosphate or deproteinized bovine bone: A systematic review and meta-analysis. Int. J. Oral Maxillofac. Surg..

[11] Yildirim M., Spiekermann H., Biesterfeld S., Edelhoff D. (2000). Maxillary sinus augmentation using xenogenic bone substitute material Bio-Oss® in combination with venous blood. Clin. Oral Implant. Res..

[12] Conz M.B., Granjeiro J.M., Gde A.S. (2005). Physicochemical characterization of six commercial hydroxyapatites for medical-dental applicatons as bone graft. J. Appl. Oral Sci..

[13] Athanasiou K.A., Zhu C.-F., Lanctot D.R., Agrawal C.M., Wang X. (2000). Fundamentals of biomechanics in tissue engineering of bone. Tissue Eng..

[14] Accorsi-Mendonça T., Conz M.B., Barros T.C., Sena L.Á.D., Soares G.D.A., Granjeiro J.M. (2008). Physicochemical characterization of two deproteinized bovine xenografts. Braz. Oral Res..

[15] Hutmacher D.W., Schantz J.T., Lam C.X.F., Tan K.C., Lim T.C. (2007). State of the art and future directions of scaffold-based bone engineering from a biomaterials perspective. J. Tissue Eng. Regen. Med..

[16] Tadic D., Epple M. (2004). A thorough physicochemical characterisation of 14 calcium phosphate-based bone substitution materials in comparison to natural bone. Biomaterials.

[17] Lu J., Descamps M., Dejou J., Koubi G., Hardouin P., Lemaitre J., Proust J.-P. (2002). The biodegradation mechanism of calcium phosphate biomaterials in bone. J. Biomed. Mater. Res..

[18] Heinz Lussi P.G. (1988). Process For Preparing High Purity Bone Mineral.

[19] Jayesh R.S., Dhinakarsamy V. (2015). Osseointegration. J. Pharm. Bioallied Sci..

[20] Lindhe J., Cecchinato D., Donati M., Tomasi C., Liljenberg B. (2014). Ridge preservation with the use of deproteinized bovine bone mineral. Clin. Oral Implant. Res..

[21] Stavropoulos A., Kostopoulos L., Mardas N., Randel Nyengaard J., Karring T. (2001). Deproteinized bovine bone used as an adjunct to guided bone augmentation: An experimental study in the rat. Clin. Implant Dent. Relat. Res..

[22] Donos N., Lang N.P., Karoussis I.K., Bosshardt D., Tonetti M., Kostopoulos L. (2004). Effect of GBR in combination with deproteinized bovine bone mineral and/or enamel matrix proteins on the healing of critical-size defects. Clin. Oral Implant. Res..

[23] Slotte C., Lundgren D. (1999). Augmentation of calvarial tissue using non-permeable silicone domes and bovine bone mineral. An experimental study in the rat. Clin. Oral Implant. Res..

[24] Suter A.J., Molteno A.C.B., Bevin T.H., Fulton J.D., Herbison G.P. (2002). Long term follow up of bone derived hydroxyapatite orbital implants. Br. J. Ophthalmol..

[25] Smith M.M., Duncan W.J., Coates D.E. (2018). Attributes of Bio-Oss((R)) and Moa-Bone((R)) graft materials in a pilot study using the sheep maxillary sinus model. J. Periodontal Res..

[26] Weiner S., Baryosef O. (1990). States of preservation of bones from prehistoric sites in the near-east—A survey. J. Archaeol. Sci..

[27] Pienkowski D., Doers T.M., Monier-Faugere M.-C., Geng Z., Camacho N.P., Boskey A.L., Malluche H.H. (1997). Calcitonin alters bone quality in beagle dogs. J. Bone Miner. Res..

[28] Landi E., Tampieri A., Celotti G., Sprio S. (2000). Densification behavior and mechanisms of synthetic hydroxyapatites. J. Eur. Ceram. Soc..

[29] Zareidoost A., Yousefpour M., Ghaseme B., Amanzadeh A. (2012). The relationship of surface roughness and cell response of chemical surface modification of titanium. J. Mater. Sci. Mater. Med..

[30] Peters F., Schwarz K., Epple M. (2000). The structure of bone studied with synchrotron X-ray diffraction, X-ray absorption spectroscopy and thermal analysis. Thermochim. Acta.

[31] Pramanik S., Hanif A.S.M., Pingguan-Murphy B., Abu Osman N.A. (2012). Morphological change of heat treated bovine bone: A comparative study. Materials.

[32] Kačarević Željka P., Kavehei F., Houshmand A., Franke J., Smeets R., Rimashevskiy D., Wenisch S., Schnettler R., Jung O., Barbeck M. (2018). Purification processes of xenogeneic bone substitutes and their impact on tissue reactions and regeneration. Int. J. Artif. Organs.

[33] Rogers K.D., Daniels P. (2002). An X-ray diffraction study of the effects of heat treatment on bone mineral microstructure. Biomaterials.

[34] Todoh M., Tadano S., Imari Y. Effect of heat denaturation of collagen matrix on bone strength. Proceedings of the 13th International Conference on Biomedical Engineering.

[35] Wang X., Bank R.A., Tekoppele J.M., Agrawal C.M. (2001). The role of collagen in determining bone mechanical properties. J. Orthop. Res..

[36] Cooper L.F., Zhou Y., Takebe J., Guo J., Abron A., Holmén A., Ellingsen J.E. (2006). Fluoride modification effects on osteoblast behavior and bone formation at TiO_2_ grit-blasted c.p. titanium endosseous implants. Biomaterials.

[37] Davies J.E. (2003). Understanding peri-implant endosseous healing. J. Dent. Educ..

[38] Shalabi M., Gortemaker A., Hof M.V., Jansen J., Creugers N. (2006). Implant surface roughness and bone healing: A systematic review. J. Dent. Res..

[39] Kim M.J., Choi M.U., Kim C.W. (2006). Activation of phospholipase D1 by surface roughness of titanium in MG63 osteoblast-like cell. Biomaterials.

[40] Ramírez Fernández M.P., Gehrke S.A., Pérez Albacete Martinez C., Calvo Guirado J.L., De Aza P.N. (2017). SEM-EDX study of the degradation process of two xenograft materials used in sinus lift procedures. Materials.

[41] Nguyen H., Morgan D.A., Forwood M.R. (2006). Sterilization of allograft bone: Effects of gamma irradiation on allograft biology and biomechanics. Cell Tissue Bank..

[42] Revenko I., Sommer F., Minh D.T., Garrone R., Franc J.M. (1994). Atomic force microscopy study of the collagen fibre structure. Biol. Cell.

[43] Kindt J.H., Thurner P.J., Lauer M.E., Bosma B.L., Schitter G., Fantner G.E., Izumi M., Weaver J.C., Morse D.E., Hansma P.K. (2007). In situ observation of fluoride-ion-induced hydroxyapatite-collagen detachment on bone fracture surfaces by atomic force microscopy. Nanotechnology.

[44] Shemesh A. (1990). Crystallinity and Diagenesis of Sedimentary Apatites. Geochim. Cosmochim. Acta.

[45] Asami A., Nakamura M., Takeuchi M., Nakayama A., Nakamura H., Yoshida T., Nagasawa S., Hiraoka B.Y., Ito M., Udagawa N. (2008). Effects of Heat Treatment of Hydroxyapatite on Osteoblast Differentiation. J. Hard Tissue Biol..

[46] Piga G., Solinas G., Thompson T., Brunetti A., Malgosa A., Enzo S. (2012). Is X-ray diffraction able to distinguish between animal and human bones?. J. Archaeol. Sci..

[47] Dal Sasso G., Asscher Y., Angelini I., Nodari L., Artioli G. (2018). A universal curve of apatite crystallinity for the assessment of bone integrity and preservation. Sci. Rep..

[48] Fleet M.E. (2013). The carbonate ion in hydroxyapatite: Recent X-ray and infrared results. Front. Biosci..

[49] Dumitrescu C.R., Neacsu I.A., Surdu V.A., Nicoara A.I., Iordache F., Trusca R., Ciocan L.T., Ficai A., Andronescu E. (2021). Nano-hydroxyapatite vs. xenografts: Synthesis, characterization, and in vitro behavior. Nanomaterials.

[50] LeGeros R.Z., Kijkowska R., Bautista C., LeGeros J.P. (1995). Synergistic effects of magnesium and carbonate on properties of biological and synthetic apatites. Connect. Tissue Res..

[51] Leeuwenburgh S., Layrolle P., Barrere F., De Bruijn J., Schoonman J., Van Blitterswijk C.A., De Groot K. (2001). Osteoclastic resorption of biomimetic calcium phosphate coatings in vitro. J. Biomed. Mater. Res..

[52] Antonia Ressler A.Ž., Ivanišević I., Kamboj N., Ivanković H. (2021). Ionic substituted hydroxyapatite for bone regeneration applications: A review. Open Ceram..

[53] Baig A.A., Fox J.L., Young R.A., Wang Z., Hsu J., Higuchi W.I., Chhettry A., Zhuang H., Otsuka M. (1999). Relationships among carbonated apatite solubility, crystallite size, and microstrain parameters. Calcif. Tissue Int..

[54] Ruppel M.E., Miller L.M., Burr D.B. (2008). The effect of the microscopic and nanoscale structure on bone fragility. Osteoporos. Int..

[55] Rammelt S., Schulze E., Witt M., Petsch E., Biewener A., Pompe W., Zwipp H. (2004). Collagen type I increases bone remodelling around hydroxyapatite implants in the rat tibia. Cells Tissues Organs.

