# Supplementary material for: The Effect of Low-Processing Temperature on the Physicochemical and Mechanical Properties of Bovine Hydroxyapatite Bone Substitutes
Source: Materials (Basel). 2022 Apr 11;15(8):2798. doi: 10.3390/ma15082798 (PMC9025514; doi:10.3390/ma15082798)
Supplement: Supplementary file 1 [file materials-15-02798-s001.zip › materials-1632143-supplementary.pdf]

materials

Supplementary Materials

The Effect of Low-Processing Temperature on the Physicochemical and Mechanical Properties of Bovine Hydroxyapatite Bone Substitutes

Dina Abdelmoneim [1,*], Gemma Claire Porter [1], Dawn Elizabeth Coates [1], Warwick John Duncan [1], John Neil Waddell [1], Niels Hammer [2,3,4] and Kai Chun Li [1]

[1] Sir John Walsh Research Institute, Faculty of Dentistry, University of Otago, Dunedin 9016, New Zealand; gemmacotton22@gmail.com (G.C.P.); dawn.coates@otago.ac.nz (D.E.C.); warwick.duncan@otago.ac.nz (W.J.D.); j.neil.waddell@icloud.com (J.N.W.); kc.li@otago.ac.nz (K.C.L.)
[2] Division of Macroscopic and Clinical Anatomy, Gottfried Schatz Research Center, Medical University of Graz 8010, Austria; niels.hammer@medunigraz.at
[3] Department of Orthopedic and Trauma Surgery, University of Leipzig 04103, Germany
[4] Fraunhofer Fraunhofer Institute for Machine Tools and Forming Technology (IWU), Medical Branch, Dresden 01187, Germany
* Correspondence: dina.abdelmoneim@otago.ac.nz

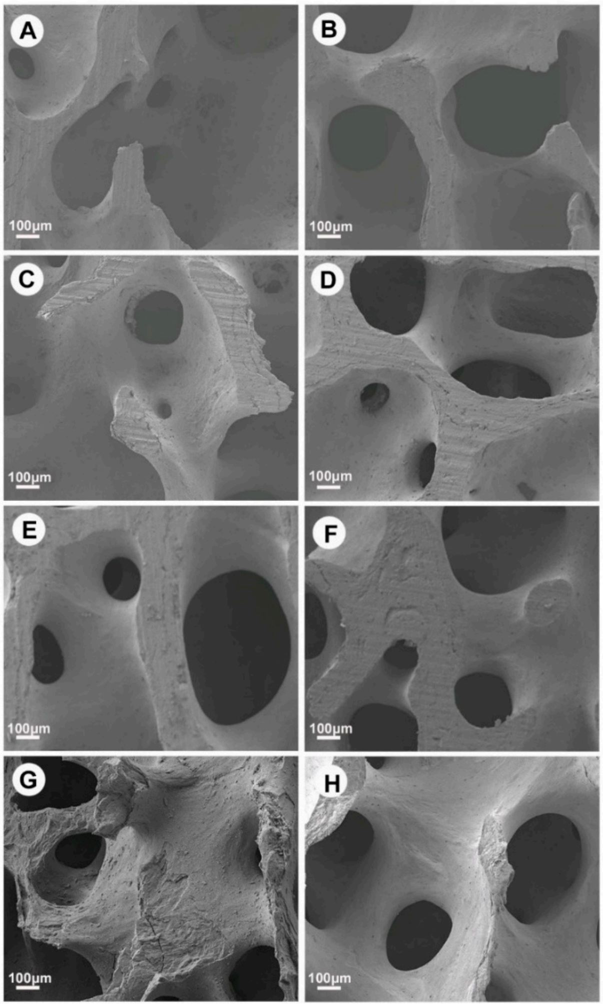

Figure S1. Representative low magnification scanning electron microscope images of the surface of heat-treated MoaBone and controls. (A) Half-processed MB, (B) MB 100 °C, (C) MB 130 °C, (D) MB 160 °C, (E) MB 190 °C, (F) MB 220 °C, (G) MoaBone®, (H) Bio-Oss®. Scale bar = 100 µm.

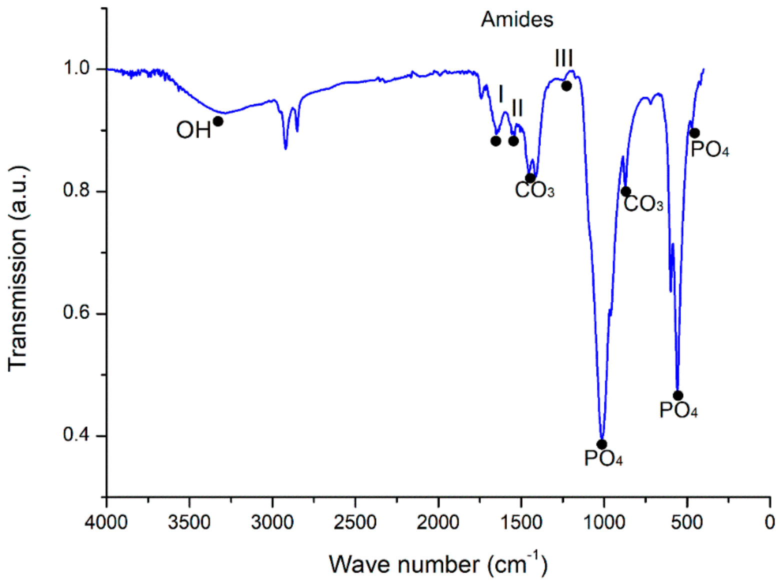

Figure S2. Representative FTIR spectra of the half-processed MoaBone with the relevant characteristic bands labelled.
